# Integrin Mediated Adhesion of Osteoblasts to Connective Tissue Growth Factor (CTGF/CCN2) Induces Cytoskeleton Reorganization and Cell Differentiation

**DOI:** 10.1371/journal.pone.0115325

**Published:** 2015-02-25

**Authors:** Honey Hendesi, Mary F. Barbe, Fayez F. Safadi, M. Alexandra Monroy, Steven N. Popoff

**Affiliations:** 1 Department of Anatomy and Cell Biology, Temple University School of Medicine, Philadelphia, Pennsylvania, United States of America; 2 Department of Anatomy and Neurobiology, Northeast Ohio Medical University (NEOMED), Rootstown, Ohio, United States of America; 3 Department of Orthopedic Surgery, Temple University School of Medicine, Philadelphia, Pennsylvania, United States of America; 4 Department of Surgery, Temple University School of Medicine, Philadelphia, Pennsylvania, United States of America; Van Andel Institute, UNITED STATES

## Abstract

Pre-osteoblast adhesion and interaction with extracellular matrix (ECM) proteins through integrin receptors result in activation of signaling pathways regulating osteoblast differentiation. Connective tissue growth factor (CTGF/CCN2) is a matricellular protein secreted into the ECM. Prior studies in various cell types have shown that cell adhesion to CTGF via integrin receptors results in activation of specific signaling pathways that regulate cell functions, such as differentiation and cytoskeletal reorganization. To date, there are no studies that have examined whether CTGF can serve as an adhesive substrate for osteoblasts. In this study, we used the MC3T3-E1 cell line to demonstrate that CTGF serves as an adhesive matrix for osteoblasts. Anti-integrin blocking experiments and co-immunoprecipitation assays demonstrated that the integrin α_v_β_1_ plays a key role in osteoblast adhesion to a CTGF matrix. Immunofluorescence staining of osteoblasts cultured on a CTGF matrix confirmed actin cytoskeletal reorganization, enhanced spreading, formation of focal adhesions, and activation of Rac1. Alkaline phosphatase (ALP) staining and activity assays, as well as Alizarin red staining demonstrated that osteoblast attachment to CTGF matrix enhanced maturation, bone nodule formation and matrix mineralization. To investigate whether the effect of CTGF on osteoblast differentiation involves integrin-mediated activation of specific signaling pathways, we performed Western blot, chromatin immunoprecipitation (ChIP) and qPCR assays. Osteoblasts cultured on a CTGF matrix showed increased total and phosphorylated (activated) forms of focal adhesion kinase (FAK) and extracellular signal-regulated kinase (ERK). Inhibition of ERK blocked osteogenic differentiation in cells cultured on a CTGF matrix. There was an increase in runt-related transcription factor 2 (Runx2) binding to the osteocalcin gene promoter, and in the expression of osteogenic markers regulated by Runx2. Collectively, the results of this study are the first to demonstrate CTGF serves as a suitable matrix protein, enhancing osteoblast adhesion (via α_v_β_1_ integrin) and promoting cell spreading via cytoskeletal reorganization and Rac1 activation. Furthermore, integrin-mediated activation of ERK signaling resulted in increased osteoblast differentiation accompanied by an increase in Runx2 binding to the osteocalcin promoter and in the expression of osteogenic markers.

## Introduction

Connective tissue growth factor (CTGF) is the second member of the CCN family of proteins which consists of six members with a similar multi-modular structure [1]. CTGF has 349 amino acids that are divided into four modules; the first module is an insulin like growth factor (IGF)-binding domain, the second is a von Willebrand type C (VWC) domain, the third is a thrombospondin-1 (TSP-1) domain, and the fourth is a C-terminal (CT) domain [2]. CTGF is considered a matricellular protein that is secreted into the extracellular matrix (ECM), where it serves as cell adhesion protein. CTGF interacts with cell surface receptors (e.g. integrins), growth factors (e.g. transforming growth factor β1 [TGF-β1]), proteases (e.g. matrix metalloproteinases [MMPs]), and ECM proteins (e.g. fibronectin), via its different modules, thereby mediating the activity of these proteins [3–5]. The multi-modular structure of CTGF and the interaction of its modules with various proteins enable CTGF to regulate a variety of cellular functions including cell adhesion, proliferation, migration, differentiation, survival, and ECM synthesis [2]. It has also been shown that CTGF is involved in more complicated biological processes such as angiogenesis, chondrogenesis, and osteogenesis, processes that are necessary for normal skeletal development [6].

The importance of CTGF in skeletogenesis was confirmed in studies utilizing mice in which CTGF is ablated. CTGF knockout mice exhibit multiple skeletal dysmorphisms, such as kinked ribs, tibiae, radii and ulnae, and craniofacial abnormalities, as a result of impaired chondrogenesis and osteogenesis [7, 8]. An in-depth characterization of the skeleton of CTGF knockout mice by our lab demonstrated numerous site-specific defects in the axial, appendicular and craniofacial skeleton [9]. Osteoblasts derived from CTGF KO mice differentiate normally *in vitro* and demonstrate a heightened response to BMP-2-induced differentiation in culture [10]. Therefore, postulate that aberrant bone development in CTGF knockout mice is not due to an intrinsic osteoblast defect but rather is secondary to defects within the bone microenvironment, including the bone matrix.

Additional studies have confirmed that osteoblasts produce and secrete CTGF during active bone formation and fracture healing [11]. Treatment of primary osteoblasts or osteoblastic cell lines (Saos-2 or MC3T3-E1) with recombinant CTGF stimulates proliferation, matrix production, mineralization, and up-regulates the expression of markers of osteoblast differentiation including type I collagen, osteopontin, osteocalcin and alkaline phosphatase [11,12]. Collectively, these studies support an anabolic role for CTGF in osteoblast differentiation and bone formation, but the mechanism responsible for this effect remains unknown.

To date, no one has identified a specific receptor for CTGF, but numerous studies have shown that integrins can serve as functional receptors for CTGF in various cell types [4,6,13–15]. This binding to integrins takes place through the third or fourth domain of CTGF [3,4,13,16], and the specific type of integrin receptor involved in CTGF binding varies based on cell type [6]. In addition to integrins, CTGF also binds to the cell surface heparan sulfate proteoglycans through its fourth domain [4]. Heparan sulfate proteoglycans serve as co-receptors for integrins and serve to strengthen the ECM protein-integrin interaction [17]. Studies have demonstrated that the CTGF-integrin interaction results in activation of signaling pathways that regulate cell differentiation, cytoskeletal reorganization and matrix production [16,18,19].

During osteoblast differentiation, mesenchymal stem cells differentiate into preosteoblastic cells and subsequently into fully functional, matrix-synthesizing osteoblasts, a process that is regulated by specific transcription factors such as Runx2 (runt-related transcription factor 2). Runx2 is downstream of the focal adhesion kinase (FAK)/extracellular signal-regulated kinase (ERK) signaling pathway [20]. Up-regulation of Runx2 expression promotes mesenchymal cells to differentiate into osteoblasts and inhibits their differentiation into adipocytes or chondrocytes [21]. In addition, Runx2 induces the expression of osteoblast-related genes such as collagen type I, fibronectin, osteopontin, bone sialoprotein, osteocalcin, and MMP-13 (matrix metalloproteinase-13) during the early stages of osteoblast differentiation [21–24].

The objective of this study is to determine whether osteoblasts can adhere to a CTGF matrix, and if so, the functional consequences of this cell/matrix interaction. We hypothesize that CTGF will serve as an effective adhesive matrix for osteoblasts and that this interaction will be mediated by one or more specific integrins expressed on the osteoblast cell surface. Furthermore, we expect this CTGF/integrin interaction will activate signaling pathways that regulate cytoskeletal reorganization and osteoblast differentiation. In the present study, we used a MC3T3-E1 preosteoblastic cell line to investigate the specific characteristics and functional consequences of osteoblast adhesion to a recombinant CTGF matrix.

## Materials and Methods

### Cell culture

MC3T3-E1 subclone 4, a mouse pre-osteoblast cell line, was purchased from ATCC (Manassas, VA). Cells were cultured in alpha minimal essential medium (Corning Cellgro, Manassas, VA) containing 10% fetal bovine serum (Atlanta Biologicals, Flowery Branch, GA) and 1% penicillin/streptomycin (Corning Cellgro). Cells were incubated at 37°C and 5% CO2.

### Western blotting

MC3T3-E1 culture dishes were washed with cold 1X PBS and cells were lysed in 1X RIPA lysis buffer (Millipore, Billerica, MA) containing 1% protease inhibitor (Sigma-Aldrich, St. Louis, MO) then incubated for 1 hour at 4°C. Protein concentration of lysates was determined using BCA Protein Assay Kit (Pierce Biotechnology, Rockford, IL). Membranes were blocked with 5% BSA/PBS- 0.1%Tween20 or LI-COR Blocking Buffer (LiCor Biosciences, St. Lincoln, NE) for 1 hour at room temperature and then incubated with the following primary antibodies: 1:500 rabbit polyclonal anti-phospho-FAK (Tyr925) (Cell Signaling Technology, Danvers, MA), 1:500 rabbit polyclonal anti FAK (Cell Signaling Technology), 1:500 rabbit monoclonal anti-phospho-ERK1/2 (Cell Signaling Technology), 1:500 rabbit monoclonal anti-ERK1/2 (Cell Signaling Technology) and 1:1000 rabbit polyclonal anti-actin (Sigma-Aldrich) overnight at 4°C. Membranes were washed with 1X PBS-0.1%Tween20 and incubated with 1:5000 horseradish peroxidase conjugated to a donkey anti-rabbit secondary antibody (Jackson Immunoresearch, West Grove, PA) or 1:5000 IRDye 800cw, donkey anti-rabbit secondary antibody from Odyssey infrared imaging system (LiCor Biosciences). Primary and secondary antibodies were diluted in blocking buffer. Bands were visualized with using a chemiluminescence detection system (Thermo Scientific, Rockford, IL) or LI-COR infrared imaging system.

### Adhesion assay

Ninety six-well non- tissue culture treated plates (Falcon Becton Dickinson, Franklin Lakes, NJ) were coated with fibronectin (Sigma-Aldrich), recombinant CTGF (ProSpec, Ness-Ziona, Israel), or 1% BSA (Fisher Scientific, Pittsburgh, PA) in PBS and were left to dry in a tissue culture hood. To block non-specific binding sites in coated wells, 1% BSA was added to the wells and the plates were incubated at 4°C for 1 hour. BSA was discarded and wells were washed with 1X PBS prior to adding 2 x10^4^ MC3T3-E1 cells (passage 5 to 10) to the wells and incubation at 37°C for 45 minutes. To block the various integrins of interest, cells were incubated with 5 μg/ml BioLegend (San Diego, CA) monoclonal integrin antibodies against α_v_, α_2_, α_5_, β_1_, β_3_, β_5_ or normal IgG for 30 minutes at 37°C prior to seeding in 96-well plates. Wells were washed with 1X PBS. CyQuant NF dye (Invitrogen, Carlsbad, Ca) was added to each well and the plates were incubated at 37°C for 1 hour. Fluorescence was measured using a microplate reader with excitation at ~485 nm and emission detection at ~530nm. Relative fluorescence units (RFUs) were converted to cell number using standard curve made by performing adhesion assay for different cell numbers.

### Proliferation Assay

Cell number was determined using the CyQUANT NF Cell Proliferation Assay Kit (Molecular Probes) according to the manufacturer’s protocol. Briefly, 1.17 x 10^4^ MC3T3-E1 osteoblastic cells were cultured on CTGF or BSA coated plates for 7 or 14 days while treated with osteogenic medium (50 μg/ml Ascorbic acid+ 10 mM β-glycerophosphate in α-MEM+ 10%FBS and 1% penicillin/ streptomycin), which was changed every 3 days. On Days 7 or 14, media was aspirated and replaced with DNA binding dye solution. Cells were incubated at 37°C for 1 hour and samples were measured using a Wallac 1420 fluorometer.

### Immunofluorescence staining

Glass chamber slides (Nalge Nunc International, Rochester, NY) were coated with 2 μg/ml recombinant CTGF (ProSpec), 2 μg/ml fibronectin (Sigma-Aldrich) or 1% BSA (Fisher Scientific) in PBS and were left to dry in a tissue culture hood. 2x10^3^ MC3T3-E1 cells were added to chambers and incubated at 37°C for 8 or 24 hours. Cells were fixed with 4% paraformaldehyde in phosphate buffer (Affymetrix, Santa Clara, CA) for 15 minutes, washed 3 times with washing buffer (1X PBS containing 0.05% Tween20) and then blocked with blocking solution (2.5% BSA in PBS) for 30 minutes at room temperature. After fixation, 1:200 primary antibody rabbit polyclonal anti-α_v_β_1_ (Bioss, Woburn, MA) and 1:500 mouse monoclonal anti-vinculin (Millipore) diluted in blocking solution, were added to the chambers, which were then incubated overnight at 4°C. After 3 washes with washing buffer, fluorescence secondary antibodies conjugated to DyLight549 or DyLight 488 (Jackson Immunoresearch) and diluted 1:2000 in blocking solution, or TRITC-conjugated phalloidin (Millipore) diluted 1:5000 in blocking solution, were added to the chambers, which were then incubated for 1 hour at room temperature. After 3 washes with washing buffer, the chamber slides were cover slipped using Vectashield mounting medium with Dapi fluorescence (Vector, Burlingame, CA). Fluorescence imaging was performed using a Nikon E1000. Cell spreading areas were measured using ImageJ.

### Rac activity assay

Rac activity assay was performed on MC3T3-E1 cells cultured for 2 hours on dishes coated with recombinant CTGF (ProSpec), fibronectin (Sigma-Aldrich), 1%BSA (Fisher Scientific), or uncoated dishes as a negative control. A Rac1 Activation Assay Biochem Kit (Cytoskeleton, Denver, CO) was used to pull down active Rac1. Then, 300 μg of total cell protein was incubated with 10 μg PAK-PBD beads, and incubated for 1 hour at 4°C. Beads were washed once with the kit’s washing buffer. After centrifugation, 20 μl of Laemmli sample buffer containing 5% β-mercaptoethanol was added to the beads and samples were boiled for 2 minutes. Western Blot analysis was performed on the samples using a mouse monoclonal anti-Rac1 antibody (Cytoskeleton).

### Co-Immunoprecipitation

When the MC3T3-E1 cells in the culture dishes were 70% confluent culture dishes, they were harvested and the cells were lysed with1X RIPA lyses buffer (Millipore) containing 1% protease inhibitor (Sigma-Aldrich). Rabbit polyclonal anti-α_v_β_1_ antibody (Bioss), rat monoclonal anti-α_2_β_1_ antibody (US BioLogical, Swampscott, MA), rat monoclonal anti-α_5_β_1_ antibody (US BioLogical), and normal IgG as a negative control (Santa Cruz Biotechnology, Santa Cruz, CA) were used to immunoprecipitate different integrin heterodimers, using the Catch and Release kit (Millipore). In spin columns, 500 μg of cell lysate, 5 μg of primary antibody or IgG and 10 μl of Antibody Capture Affinity Ligand were incubated at room temperature for 30 minutes. Spin columns were centrifuged, the flow-through was discarded, and the beads in columns were washed 3 times with the kit’s washing solution. To elute the protein of interest, 1X Denaturing Elution Buffer containing β-mercaptoethanol was added to columns and heated at 97°C for 3 minutes. Denatured proteins were collected by centrifugation. For Western blot analysis, goat polyclonal anti-CTGF antibody (Santa Cruz Biotechnology) or rabbit polyclonal anti-β_1_ integrin (Cell Signaling) diluted 1:200 in blocking buffer, and then a horseradish peroxidase conjugated donkey anti- goat or donkey anti-rabbit secondary antibody (Jackson Immunoresearch) diluted 1:5000 in blocking buffer were applied, as described previously.

### Flow cytometry

Cells were detached using 10mM EDTA in PBS and gentle scraping followed by centrifugation. After washing once in PBS, cells were resuspended in PBS containing 4% formaldehyde and fixed for 10 minutes at 37°C. After fixation, cells were chilled on ice and washed twice with PBS. In each assay tube, 1x10^6^ cells were incubated with 1/100 primary antibody (α_v_, α_2_, α_5_, β_1_, β_3_ or β_5_ [BioLegend]) or isotype IgG in buffer solution (0.5 g BSA in 100 ml 1XPBS) for an hour at room temperature. Cells were washed and then resuspended in 1/5000 secondary antibody (DyeLight 488, Alexa Fluor 647 [Jackson ImmunoResearch]) in buffer solution for 30 minutes a room temperature. Cells were washed, resuspended in 1ml of buffer solution, and used for flow cytometric analysis on a Calibur (BD Biosciences) analyzer.

### Alkaline phosphatase staining and activity

Forty eight-well plates (Falcon Becton Dickinson) were coated with 2 μg/ml recombinant CTGF (ProSpec) or 1% BSA (Fisher Scientific) in PBS. 1.17 x 10^4^ MC3T3-E1 cells suspended in α-MEM containing 10%FBS and 1% penicillin/streptomycin were seeded in each well. At day 3 of culture, cells were treated with osteogenic medium (50 μg/ml Ascorbic acid+ 10 mM β-glycerophosphate in α-MEM+ 10%FBS and 1% penicillin/ streptomycin), which was changed every 3 days. In the case of ERK inhibitor, 10 μM U0126 MEK1/2 inhibitor (Cell Signaling) was added to the osteogenic medium. Alkaline phosphatase staining was performed on day 14 of culture using a Leukocyte Alkaline Phosphatase kit and its solutions (Sigma-Aldrich). After washing cells in 1X HBSS, they were fixed in citrate buffer containing acetone and formaldehyde. After fixation, cells were washed with ddH2O and stained with a solution containing sodium nitrate, FRV-alkaline solution and napthol AS-BI alkaline solution for 25 minutes at room temperature. Cells were washed with ddH2O, air-dried and images were taken using a Nikon Eclipse TE300 inverted microscope.

Alkaline phosphatase activity was quantified using a Quantichrome Alkaline Phosphatase kit (BioAssays Systems, Hayward, CA). On day 14 of culture, cells from additional cultures were digested with ddH2O + 0.2% Triton X-100 and incubated at room temperature for 20 minutes. Cells were scraped, centrifuged, supernatant collected and assay was performed according to the manufacturer’s protocol. Alkaline phosphatase activity was normalized to total amount of protein, determined using a BCA assay kit (Pierce Biotechnology).

### Alizarin red staining

MC3T3-E1 osteogenic cells were cultured on recombinant CTGF (ProSpec) or 1% BSA (Fisher Scientific) coated 48-well plates (Falcon Becton Dickinson) and terminated on day 35 to evaluate osteoblast mineralization. Cells were washed with ddH_2_O and fixed with 10% paraformaldehyde in phosphate buffer for 15 minutes at room temperature. After washing cells with 1X PBS, 40 mM Alizarin red stain (Sigma-Aldrich) was added to wells and incubated at room temperature for 15 minutes. Cells were washed with ddH_2_O and air-dried. Images were taken using a Nikon Eclipse TE300 inverted microscope. ImageJ software was used to measure nodule area.

### Chromatin immunoprecipitation (ChIP) assay

ChIP assay was performed on MC3T3-E1 cells cultured on recombinant CTGF (ProSpec) coated, 1%BSA (Fisher Scientific) coated or un-coated culture dishes, and treated for 7 days with 50 μg/ml Ascorbic acid+ 10 mM β-glycerophosphate in α-MEM+ 10%FBS and 1% penicillin/streptomycin. Cultured cells were cross-linked with 1% paraformaldehyde (Affymetrix) in phosphate buffer for 10 minutes at room temperature. Cells were scraped and collected in 1% PBS, centrifuged, and pellets were resuspended in lysis buffer (50mM Hepes-KOH [pH 8.0], 1mM EDTA, 140mM NaCl, 10% Glycerol, 0.5% NP-40, 0.25% Triton X-100, 1mM PMSF) containing 1% protease inhibitor (Sigma-Aldrich) and incubated at room temperature for 10 minutes. Samples were centrifuged and pellets were resuspended in dilution buffer (10mM Tris-HCl [pH 8.0], 1% triton X-100, 0.1% SDS, 1mM EDTA, 140 mM NaCl, 1mM PMSF and 1% protease inhibitor). Lysates were sonicated 8 times, each time for 10 seconds at 4°C. After high-speed centrifugation (10 minutes, 13,000 x g), 25μl of supernatants were stored for input of ChIP reaction. The remainder of the supernatants were incubated with protein A/G-agarose beads (Santa Cruz Biotechnology) for 1 hour at 4°C to pre-clear samples. Runx2 antibody (Cell Signaling Technology), Acetyl-Histone4 antibody (Millipore) as a positive control and normal rabbit IgG (Santa Cruz Biotechnology) as a negative control were added to pre-cleared supernatants and incubated overnight at 4°C. Immuno-complexes were isolated by adding 60 μl of protein A/G-agarose beads, incubated for 1 hour at 4°C, then centrifugation for 1 minute at 2,000 x g. The beads were washed with low salt immune complex wash buffer (0.1% SDS, 1% Triton X-100. 2 mM EDTA, 20 mM Tris-HCl [pH 8.1], 150 mM NaCl), high salt immune complex wash buffer (0.1% SDS, 1%Triton X-100, 2 mM EDTA, 20 mM Tris-HCl [pH 8.1], 500 mM NaCl), LiCl immune complex wash buffer (0.25 M LiCl, 1% IGEPAL-CA630, 1% deoxycholic acid (sodium salt), 1mM EDTA, 10mM Tris, [pH 8.1]) and TE buffer (10mM Tris-HCl, 1mM EDTA, [pH 8.0]). The immunocomplexes were eluted by resuspending the beads in 500 μl of elution buffer (1% SDS, 0.1 M NaHCO3 [pH 8.0]). After centrifugation, supernatants were collected, 20 μl of 5M NaCl was added to reverse cross-links, and samples were heated overnight at 65°C. Next, 10 μl of 1M Tris-HCl [pH 6.5], 10 μl of 0.5 M EDTA [pH 8.0], and 2 μl of 10mg/ml proteinase K (Sigma) were added to the eluates, which were incubated for 1 hour at 45°C. DNA fragments were extracted with 500 μl of phenol chloroform (Acros Organics) and centrifuged for 3 minutes at maximum speed at room temperature. Following centrifugation, 50 μl of 3 M potassium acetate [pH 5.5], 2 μl Glycogen glycogen (Roche, Basel Switzerland) and 1 ml 100% ethanol were added to the samples and incubated on dry ice for 2 hours. Samples were centrifuged and pellets were washed with 70% ethanol and resuspended in ddH20. DNA that was isolated from the immuno-complexes and DNA from the input (25 μl of lysate after sonication) were subjected to Quantitative PCR. A standard curve of known target DNA was constructed in parallel from which the relative amount of target DNA in the sample was calculated. Control ChIP assays with normal IgG were performed for each experiment.

The quantification of ChIP-DNA by quantitative PCR was performed using primers from a previous study [25]. For detection of the Runx2 binding site on the mouse osteocalcin gene promoter (OSE2b; -611/-605) F’: TGCCTCCATAGATCCGGTT and R’: CCCACAATGGGCTAGGCTC, and for negative control primer that does not produce a PCR product with ChIP-DNA, F’: CTGCCAGGCTTCCTGCTAGT and R’: TACAGATGCCAAGCCCAGC were used. OSE2b promoter occupancy by Runx2 transcription factor was determined using Syber Green Master Mix (Applied Biosystems), loading equal amount of DNA, and running cycles of 2 min at 50°C, 10 min at 95°C, and 40 cycles of 15s at 95°C and 1 min at 60°C. PCR was performed using an Applied Biosystems 7500 Real-time PCR System SDS v1.2.

### RNA Isolation and Quantitative PCR

Total RNA was isolated from MC3T3-E1 cells cultured on CTGF coated or BSA coated plates for 7 days. RNA isolation was performed using Trizol reagent (Invitrogen) according to the manufacturer’s directions. RNA was purified using the RNeasy Mini Kit (Qiagen) and treated with DNase using the RNase-Free DNase Kit (Qiagen). RNA quality and quantity was determined using spectrophotometry and the integrity of all samples was confirmed using 1% formaldehyde-agarose gel. For cDNA synthesis, 1 μg of cDNA was transcribed from total RNA using SuperScript First-Strand Synthesis System (Invitrogen). Gene expression for runt-related transcription factor 2 (*Runx2*), alkaline phosphatase (*Alp*), and osteocalcin (*Oc*) was determined by qPCR, using the identical primers as described previously [9].

### Statistical analysis

One-factor analysis of variance (ANOVA) was performed to evaluate the effect of each variable on two or more independent groups. In the event of a significant group effect, a Bonferroni post-test was performed to compare selected pairs of group means. Adjusted p values are reported. For comparisons between two group means in which the response was affected by a single variable, an unpaired *t*-test was performed. Any difference with a probability value less than 0.05 was considered statistically significant.

## Results

### CTGF as a substrate for integrin-dependent osteoblast adhesion

**Fig 1:**
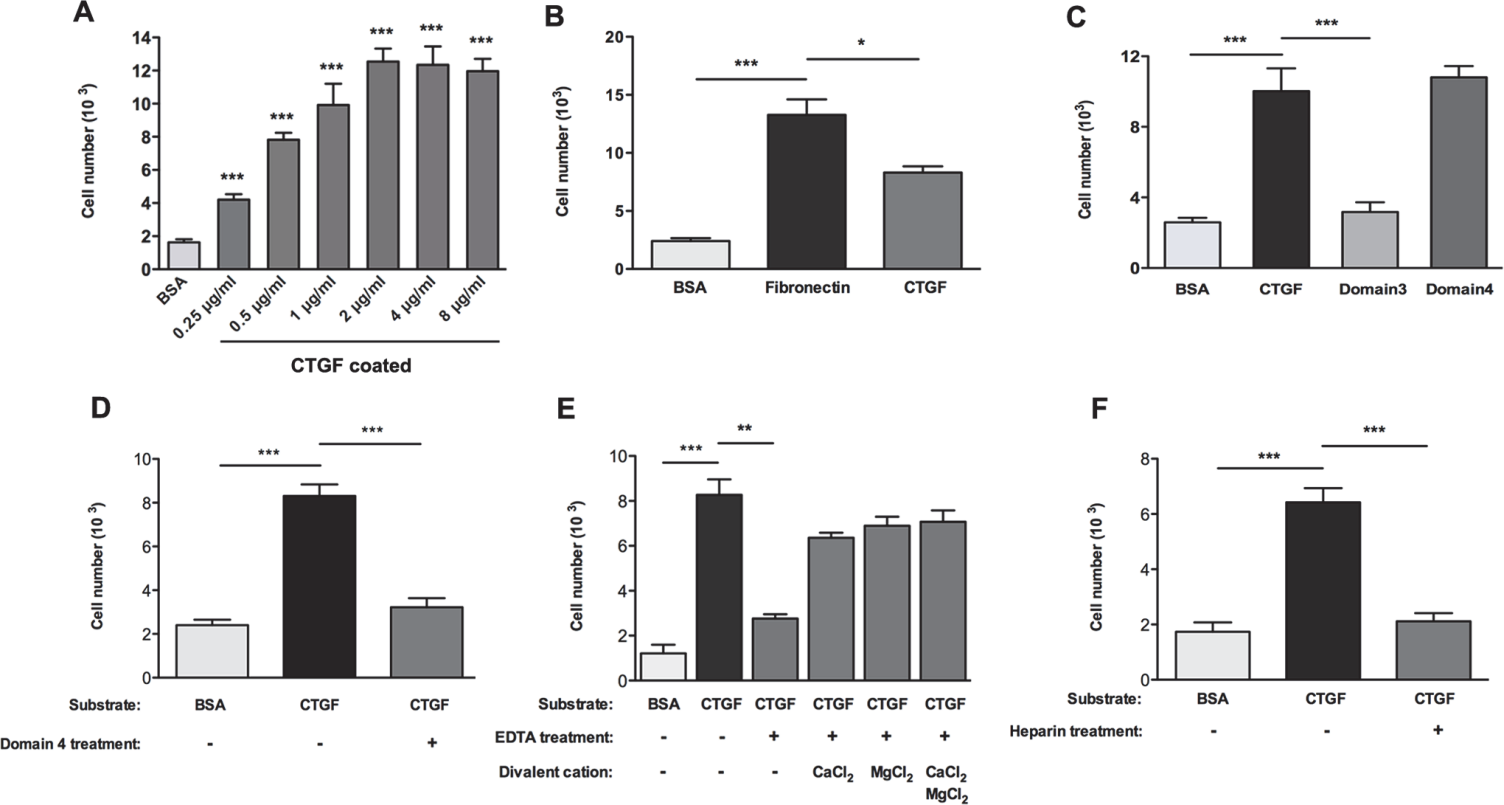
Osteoblast adhesion to CTGF is via integrin receptors and through fourth domain of CTGF. **(A)** Adhesion assay to analyze osteoblast adhesion to various concentrations of recombinant CTGF, used to coat the wells. The number of cells adhered to CTGF is compared to cell adhesion to 1% BSA (negative control). **(B)** Adhesion assay comparing osteoblast adhesion to 2 μg/ml CTGF, 2 μg/ml fibronectin, or 1% BSA (negative control) coated wells. **(C)** Adhesion assay comparing osteoblast adhesion to 2 μg/ml of full-length CTGF, third domain of CTGF, fourth domain of CTGF, or 1% BSA (negative control) coated wells. **(D)** Adhesion assay analyzing effect of osteoblast treatment with fourth domain of CTGF prior to culture on full-length CTGF coated wells. **(E)** Adhesion assay analyzing effect of divalent cation chelation by EDTA on osteoblast adhesion to full-length CTGF, and reversing this effect by adding excessive amount of divalent cations to the culture. **(F)** Adhesion assay studying the effect of blocking heparin binding site of CTGF molecule via adding heparin to osteoblast cell suspension prior to culture on CTGF coated wells. n = 6, *p<0.05; **p<0.01; ***p<0.001. Adhesion assays were repeated three times with similar results.

To evaluate whether CTGF can serve as a matrix for osteoblast attachment, we conducted an adhesion assay using MC3T3-E1 osteoblasts that were seeded onto culture plates coated with various concentrations of CTGF. This assay demonstrated a significant, concentration-dependent increase in the attachment of cells plated onto the CTGF substrate (ranging from 0.25 to 2 μg/ml) compared to BSA (negative control), with cell attachment reaching a plateau at 2 μg/ml CTGF (Fig. 1A). Next, we compared the attachment of osteoblasts to CTGF and fibronectin, a well-documented bone matrix protein that promotes osteoblast adhesion [26–28]. While osteoblasts adhered to both fibronectin and CTGF (2 μg/ml), the number of cells that attached to fibronectin was greater than to CTGF under identical assay conditions (Fig. 1B). Next, we investigated which domain of CTGF provides the binding site for osteoblast adhesion. For these experiments, we coated wells with the third domain of CTGF, the fourth domain of CTGF, or the full length CTGF protein and performed adhesion assays. Osteoblast adhesion to the fourth domain was comparable to adhesion to the whole CTGF protein (Fig. 1C), while adhesion to the third domain was not significantly different from BSA (the negative control). To further determine if the fourth domain of CTGF plays an essential role for osteoblast adhesion to CTGF, we incubated osteoblasts with recombinant domain four for 30 minutes at room temperature prior to seeding cells in wells coated with full length CTGF. Pre-incubation of cells with the fourth domain of CTGF blocked adhesion to full length CTGF with levels comparable to the negative control (BSA) (Fig. 1D).

Previous studies have demonstrated that integrins serve as functional receptors for the adhesion of various cell types to CTGF [6]. To examine whether the attachment of osteoblasts to CTGF was integrin-dependent, we examined two characteristics of integrin-dependent adhesion, namely the requirement of divalent cations for integrin activation and ligand binding [29], and the role of heparan sulfate proteoglycans as integrin co-receptors in cell adhesion [17]. Treatment of cells with 5 mM EDTA chelated the divalent cations and impaired cell adhesion (Fig. 1E). When an excess amount of divalent cations (10 mM of MgCl_2_ or CaCl_2_) were added back to the EDTA treated cell suspensions, cell adhesion was restored (Fig. 1E). Next, we conducted an adhesion assay in which 0.1 μg/ml of heparin was added to the cell suspension. The heparin was bound to the heparin- binding site (within the fourth domain) of CTGF, thereby making it unavailable for cell surface heparan sulfate proteoglycans to bind to CTGF [30,31]. Heparin treatment significantly impaired osteoblast adhesion to CTGF (Fig. 1F). Collectively, these adhesion assays demonstrate that CTGF can serve as a substrate for osteoblast adhesion and that the adhesive interaction is likely to involve the binding of cell surface integrins (as receptors) and heparan sulfate proteoplycans (as co-receptors) to the fourth domain of CTGF.

### Integrin α_v_β_1_ is the primary receptor mediating osteoblast adhesion to CTGF

**Fig 2:**
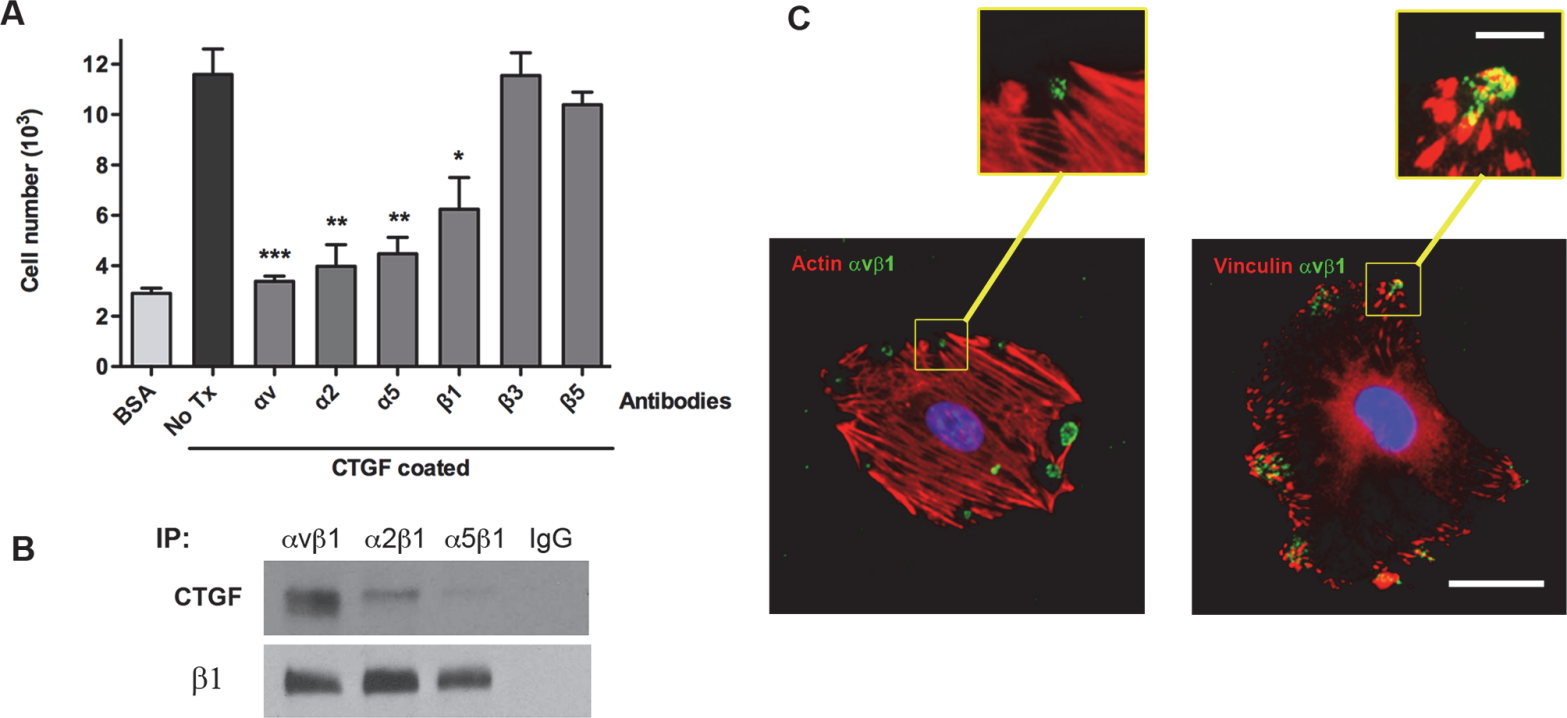
Osteoblast adhesion to CTGF is mediated by α_v_β_1_ integrin receptor. **(A)** Adhesion assay of osteoblasts treated with different blocking integrin antibodies prior to culture on CTGF or BSA (negative control) coated wells. The number of adhered cells in each treatment group was compared to the adhesion level of untreated cells. Adhesion to CTGF matrix after treatment with β_3_ or β_5_ integrin antibodies was not significantly different from untreated cells. **(B)** Western blot analyzing CTGF levels following immunoprecipitation of different integrin heterodimers from osteoblast cell lysates and control blot analyzing β_1_ integrin levels to confirm that comparable amounts of integrin heterodimers were pulled down during the immunoprecipitation using their respective antibodies. IgG was used as negative control for integrin antibodies. **(C)** Immunofluorescence staining of osteoblasts cultured on CTGF coated slides for 24 hours at 37°C. Cells stained for α_v_β_1_ (green), F-actin (red in left panel), or vinculin (red in right panel). Lower panel: Scale bar = 50 μm. Upper panel: Scale bar = 10 μm. n = 6, *p<0.05; **p<0.01; ***p<0.001. Experiments were repeated three times with similar results.

To determine which integrin mediates osteoblast adhesion to CTGF, we used blocking antibodies directed against the six primary integrin monomer subunits expressed on osteoblasts. Cells were incubated with 20 μg/ml of the appropriate blocking antibody for 30 minutes at 37°C prior to performing the adhesion assay. We observed a significant decrease in osteoblast adhesion to CTGF when α_v_, α_2_, α_5_, or β_1_ integrins were blocked, while blocking β_3_ or β_5_ integrins did not have any effect on osteoblast adhesion (Fig. 2A). To further examine the specificity of the CTGF-integrin interaction in osteoblasts, we immunoprecipitated different integrin heterodimers (α_v_β_1_, α_2_β_1_, α_5_β_1_) from osteoblast cell lysates and examined CTGF levels in these integrin pull downs by Western blotting. These analyses confirmed that the most significant interaction occurs between the α_v_β_1_ integrin and CTGF (Fig. 2B). In addition, we conducted flow cytometry which demonstrated highest levels of expression of the αv and β1 integrin subunits compared to the other α and β subunits (S1 Fig.). Immunofluorescent staining of osteoblasts cultured on recombinant CTGF demonstrated actin stress fibers, clustering of α_v_β_1_ integrin receptors and vinculin staining in the areas of focal adhesions (Fig. 2C). The actin stress fibers converged on the sites of focal adhesions, and α_v_β_1_ integrin and vinculin staining was co-localized in these areas (Fig. 2C, insets). Collectively, these data suggest that α_v_β_1_ is the primary integrin involved in osteoblast adhesion to the CTGF matrix, but they do not preclude that α_2_β_1_ and α_5_β_1_ may also play a role, albeit to a lesser extent, in this process.

### Adhesion to CTGF promotes cytoskeletal reorganization, cell spreading and Rac activation in osteoblasts

**Fig 3:**
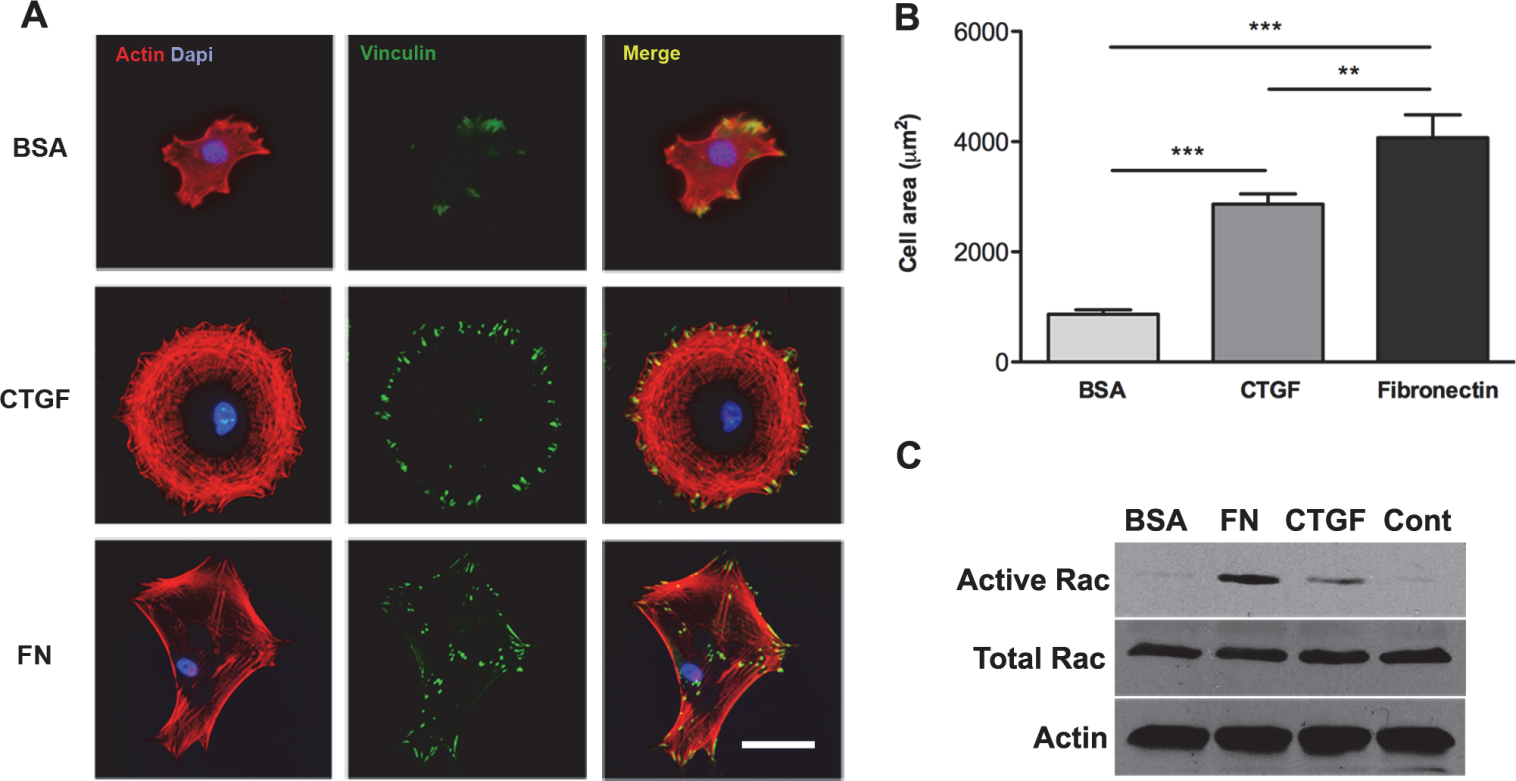
Osteoblast adhesion to CTGF matrix induces Rac activation and cell spreading. **(A)** Immunofluorescence staining of F-actin in osteoblasts cultured on 1% BSA, 2 μg/ml of CTGF or fibronectin coated slides for 8 hours at 37°C. Scale bar = 50 μm. **(B)** Cell spreading area of osteoblasts cultured on BSA, CTGF or fibronectin for 8 hours and stained for actin were measured by ImageJ; n = 50. **p<0.01; ***p<0.001. **(C)** Western blot analysis of active Rac1, total Rac1 and actin to study Rac1 activation levels. Osteoblasts were cultured for 2 hours on uncoated plates or plates coated with BSA, CTGF or fibronectin. Abbreviations: fibronectin (FN) and negative control (Cont). Experiments were repeated three times with similar results.

Regulation of cytoskeletal reorganization is an important aspect of integrin dependent cell adhesion to extracellular matrix proteins [32]. To investigate the effects of osteoblast adhesion to CTGF on cytoskeletal reorganization and cell spreading, we compared cells that were cultured for eight hours on substrates of CTGF, BSA (negative control) or fibronectin (positive control) (Fig. 3A and B). Immunofluorescence staining of actin filaments and vinculin demonstrated that cells attached to CTGF had a more uniform, rounded shape, compared to cells attached to fibronectin, which exhibited a more polarized appearance (Fig. 3A). It is interesting to note that this difference in shape was more pronounced at earlier time points; many of the cells on CTGF had a more polarized appearance by 24 hours (see Fig. 2C). Quantification of cell area demonstrated that cells plated on CTGF spread well, compared to BSA, but not to the same extent as cells on fibronectin (Fig. 3B). We next examined if osteoblast adhesion/spreading on CTGF results in the activation of Rac1, a key signaling molecule in the regulation of cell spreading [33,34]. We performed Rac1 activation assays on cells cultured on dishes coated with BSA, fibronectin, or CTGF or on uncoated dishes for 2 hours. Activation of Rac1 occurred in cells cultured on fibronectin and CTGF compared to BSA or uncoated controls, with highest levels of active Rac1 observed in cells cultured on fibronectin (Fig. 3C). Total Rac levels were similar in under all conditions (Fig. 3C). Collectively, these data support a role for CTGF as matrix protein that promotes cytoskeletal reorganization, spreading and Rac activation in osteoblasts.

### CTGF matrix enhances osteoblast differentiation and matrix mineralization

**Fig 4:**
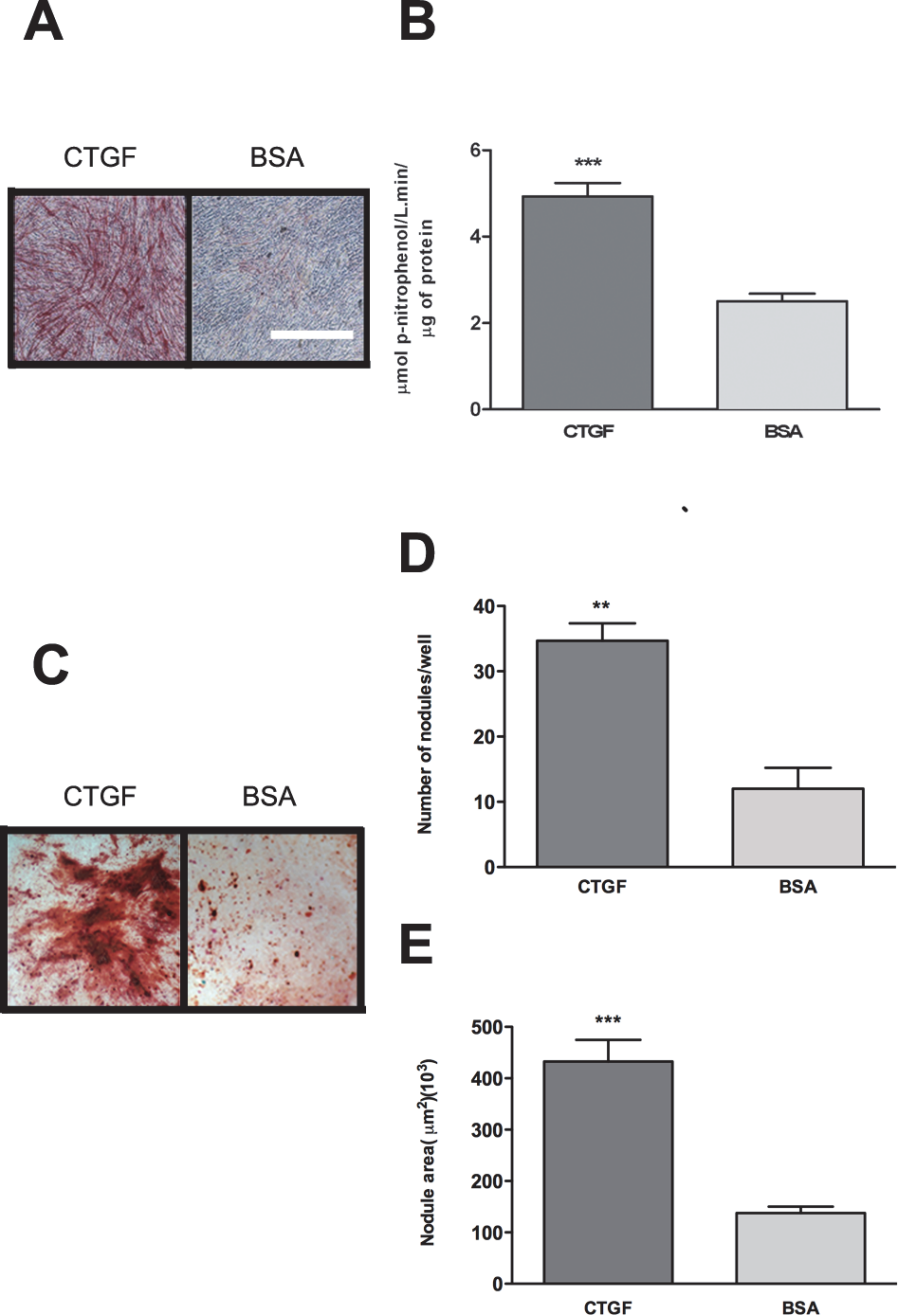
Osteoblast adhesion to CTGF matrix enhances osteoblast maturation and matrix mineralization. **(A)** Alkaline Phosphatase (ALP) staining of osteoblasts cultured on 2 μg/ml CTGF or 1% BSA coated plates for 14 days. Scale bar = 2 mm. **(B)** ALP activity quantified at day 14 of culture and normalized to total protein content; n = 9 wells. **(C)** Alizarin red staining of osteoblasts cultured on 2 μg/ml CTGF or 1% BSA coated plates for 35 days. Same magnification as in A. **(D)** Number of nodules formed after 35 days of culture; n = 9 wells. **(E)** Area of nodules measured by ImageJ software. **p<0.01; ***p<0.001. Experiments were repeated three times with similar results.

Previous *in vivo* and *in vitro* studies have demonstrated a role of CTGF in osteogenesis and early stages of osteoblast differentiation. To examine the effect of osteoblast adhesion to a CTGF matrix on subsequent differentiation, we cultured preosteoblastic cells (MC3T3-E1) on a matrix of CTGF or BSA (negative control) coated plates under osteogenic conditions (see Materials and Methods for details). After 14 days, we assessed osteoblast maturation by alkaline phosphatase (ALP) staining (Fig. 4A) and activity (Fig. 4B). The results demonstrated a more robust staining for ALP and significantly higher enzyme activity in cells cultured on CTGF compared to BSA (Fig. 4A and B). To address whether the increased differentiation observed when cells are cultured on a CTGF substrate could be an indirect effect of increased cell proliferation, we performed a proliferation assay. Cells cultured on CTGF-coated and BSA-coated plates did not exhibit a significant difference in cell number at days 7 or 14 (S2 Fig.). From these results we concluded that increased differentiation is not secondary to increased numbers of adhered/proliferating osteoblasts on CTGF substrate. Next, we evaluated bone nodule formation and matrix mineralization at day 35 of culture. Staining for Alizarin red showed large, well-formed nodules in cells cultured on the CTGF matrix compared to BSA (Fig. 4C). The number (Fig. 4D) and area (Fig. 4E) of nodules were also significantly greater on CTGF compared to BSA. Collectively, these data demonstrate that preosteoblasts grown on a CTGF matrix results in enhanced osteogenic differentiation, bone nodule formation and matrix mineralization.

### Osteoblasts cultured on a CTGF matrix activate an important osteoblast differentiation signaling pathway

**Fig 5:**
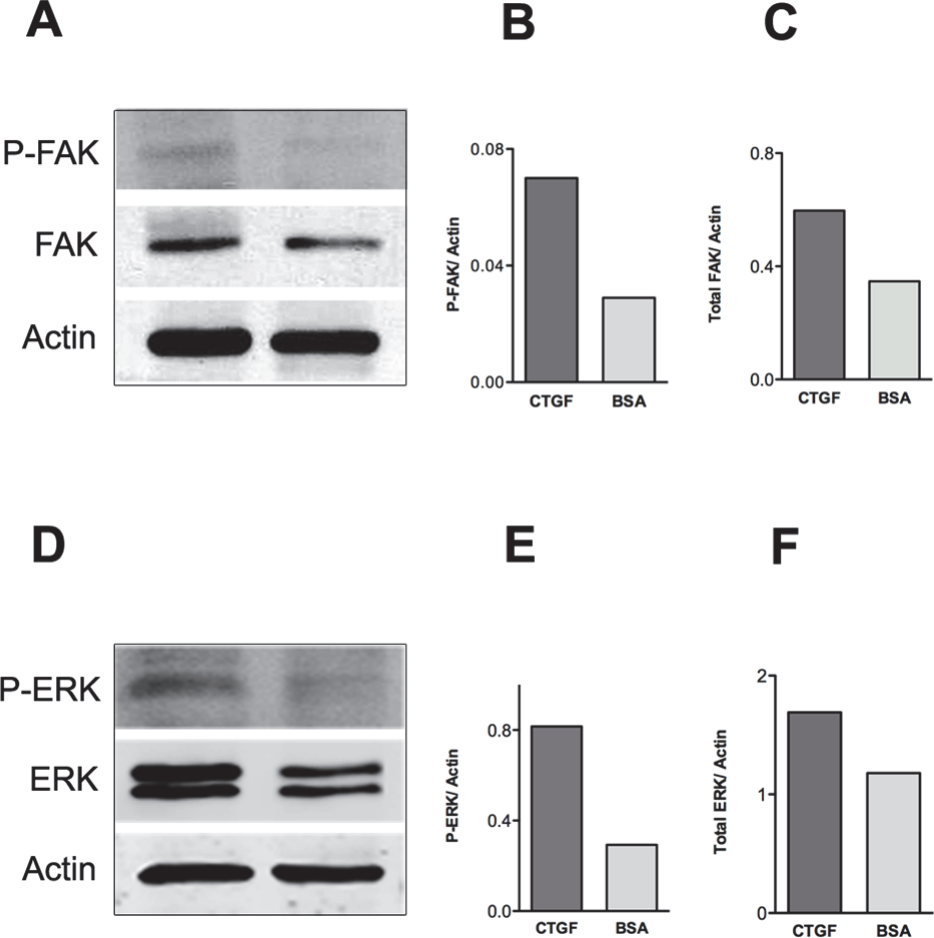
Osteoblast adhesion to CTGF matrix induces FAK, ERK and Runx2 activation. **(A)** Western blot analysis of p-FAK, total FAK and actin protein levels at day 7 of osteoblast culture on 2 μg/ml CTGF or 1% BSA coated plates while treated with osteogenic medium. **(B)** P-FAK levels were normalized to actin. **(C)** Total FAK levels were normalized to actin. **(D)** Western blot analysis of p-ERK, total ERK and actin protein levels at day 7 of osteoblast culture on 2 μg/ml CTGF or 1% BSA coated plates. **(E)** P-ERK levels were normalized to actin. **(F)** Total ERK levels were normalized to actin. All Western blots were repeated a minimum of three times with similar results.

The MAPKinase signaling pathway (including FAK and ERK1/2), which activates Runx2, is a well-known signaling pathway leading to osteoblast differentiation [20]. To examine if osteoblasts cultured on CTGF results in activation of these signaling molecules, we cultured cells on CTGF or BSA (negative control) for a period of 7 days and compared expression of activated and total FAK and ERK1/2 proteins (Fig. 5A-F). Western blot analyses demonstrate that osteoblasts on CTGF expressed higher levels of both activated and total FAK and ERK (Fig. 5A-F). We also performed Western blots to examine the short term activation of ERK after seeding onto CTGF, and observed a rapid (within 30 minutes) increase in p-ERK activation (S3 Fig.).

**Fig 6:**
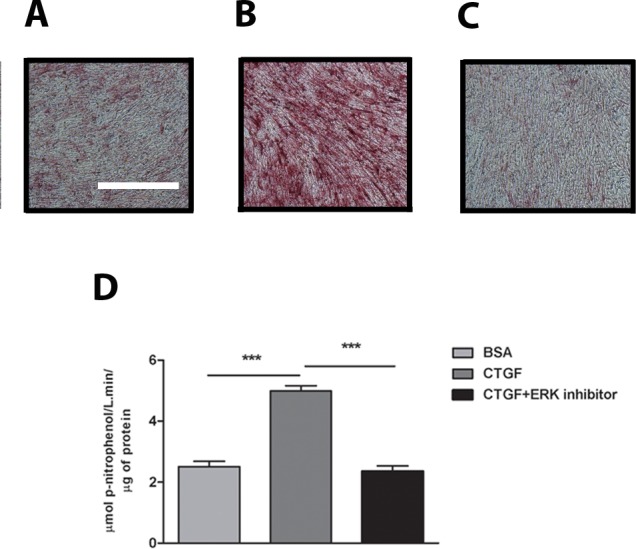
Inhibition of ERK prevents osteogenic differentiation in cells cultured on CTGF matrix. Alkaline Phosphatase (ALP) staining of osteoblasts cultured on 1% BSA **(A)** or 2 μg/ml CTGF coated plates in the absence of ERK inhibitor **(B)** and in the presence of ERK inhibitor **(C)** for 14 days. Scale bar = 2 mm. **(D)** ALP activity of osteoblasts cultured on BSA or CTGF coated plates, quantified at day 14 of culture and normalized to total protein content; n = 9 wells.

To confirm that activation of the ERK pathway plays a central role in CTGF induced osteoblast differentiation, we performed additional experiments using cells cultured on CTGF-coated plates with and without the ERK inhibitor, U0126. In these experiments, we assessed ALP staining and activity after 14 days in culture using BSA-coated plates as our negative control. Inhibition of ERK completely blocked the increased ALP staining and activity observed when cells are cultured on CTGF-coated plates (Fig. 6).

**Fig 7:**
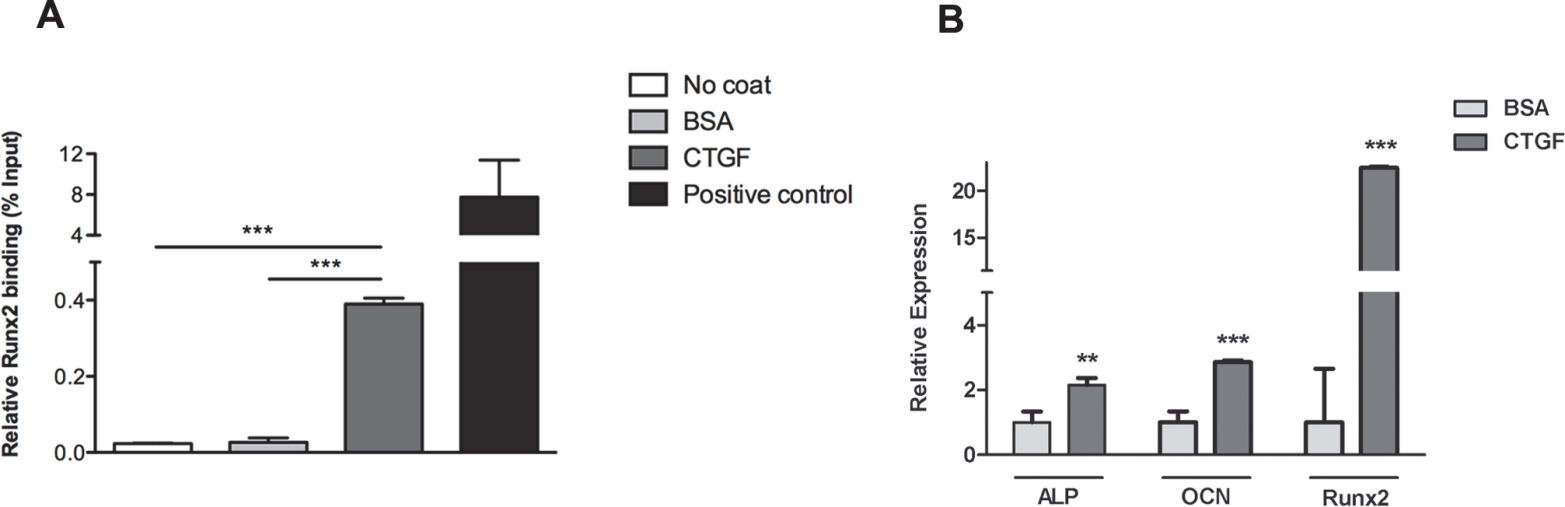
Osteoblast adhesion to CTGF induces Runx2 transcriptional activation of osteogenic markers. **(A)** ChIP assay performed on osteoblasts cultured on CTGF coated, BSA coated or uncoated plates for 7 days while treated with osteogenic medium. Runx2 antibody or acetyl-Histone H4 antibody (positive control) used for chromatin immunoprecipitation. Quantitative PCR was performed using osteocalcin gene promoter primers. n = 3, ***p<0.001. ChIP assay repeated three times with similar results. **(B)** Quantitative PCR performed on osteoblasts cultured on CTGF or BSA coated plates for 7 days while treated with osteogenic media. OCN = osteocalcin, ALP = alkaline phosphatase. n = 3, **p<0.01; ***p<0.001. Experiments repeated three times with similar results.

Runx2 is a critical transcription factor for osteoblast differentiation that has been shown to regulate the transcription of other essential proteins such as osteocalcin [21,35]. To investigate if osteoblasts cultured on a CTGF matrix have an effect on Runx2 transcriptional activity, we performed chromatin immunoprecipitation (ChIP) assays to analyze Runx2 binding occupancy on the osteocalcin gene promoter (Fig. 7A). Cells cultured on CTGF for 7 days showed a significant increase in Runx2 binding occupancy compared to cells on BSA or uncoated plates (Fig. 7A). Next, we performed a quantitative PCR analysis to compare expression of osteogenic markers (Runx2, ALP and osteocalcin) in osteoblasts cultured on CTGF matrix for 7 days (the same condition as ChIP assay). We detected significant increases in the expression of each of these osteogenic markers in osteoblasts were cultured on CTGF-coated as compared to BSA-coated plates (Fig. 7B). Collectively, these data demonstrated that when osteoblasts are cultured on CTGF, there is a sustained up-regulation and activation of FAK and ERK, increased Runx2 binding occupancy of the osteocalcin gene promoter, and increased expression of osteogenic markers that are regulated by Runx2. These results also confirmed that CTGF induced activation of ERK signaling is necessary for osteoblast differentiation, consistent with previous reports regarding the central role of ERK signaling in osteoblast differentiation [20].

## Discussion

In this study, we investigated the properties of osteoblast adhesion to a CTGF matrix and the functional consequences of this interaction. We demonstrated that osteoblasts attach to CTGF in a concentration dependent manner, that the α_v_β_1_ integrin is the primary osteoblast receptor for CTGF, and that the binding site for osteoblast adhesion is contained within the fourth domain of CTGF. We showed that osteoblasts spread well on a CTGF matrix through activation of Rac1 and they form focal adhesions containing clusters of integrins that can activate the FAK/ERK signaling pathway. Osteoblasts cultured on a CTGF matrix demonstrated enhanced bone nodule formation and matrix mineralization, and increased osteogenic differentiation was accompanied by increased Runx2 binding occupancy of the osteocalcin promoter.

Cell attachment is mediated by direct ligation of the integrin extracellular domains with defined sequences within ECM proteins [36]. Most of our current knowledge of CTGF-integrin interactions comes from studies using non-skeletal cells [6]. CTGF serves as both an adhesive substrate for cells, and a molecular bridge between other ECM proteins, such as fibronectin [3]. This adhesive process is sufficient to induce distinct cellular responses consistent with CTGF being a secreted, ECM-associated protein that exhibits remarkable functional diversity, influencing cell proliferation, migration/chemotaxis, differentiation, survival and matrix production in various different cell types [19, 37–41].

Our study reveals some important aspects of the interaction between osteoblast cell surface integrins and CTGF. We demonstrated that the β_1_ integrin subunit is critical for osteoblast adhesion to CTGF. Based on the relative amount of CTGF that bound to α_v_β_1_ compared to α_2_β_1_ or α_5_β_1_, as well as the relative levels of expression of the α_v_ and β_1_ integrin subunits compared to the other α and β subunits, α_v_β_1_ appears to be the primary integrin heterodimer involved in osteoblast adhesion to CTGF. The integrins α_2_β_1_ and α_5_β_1_ may also play a role, although to a lesser extent, in this process. We also demonstrated that HSPGs act as co-receptors to promote the osteoblast integrin-CTGF adhesive interaction. This finding is consistent with integrin-CTGF interactions described in other cell types [4,30]. Two members of the HSPG family, namely Syndecan 1 and 4, have been shown to localize to sites of cell-matrix adhesion where they have been shown to play a synergistic role with cell surface integrins in mediating cell adhesion [42,43].

At first glance, one may expect that blocking the β_1_ integrin that complexes with α_v_, α_2_, or α_5_ should abolish the adhesion of all α_v_β_1_, α_2_β_1_, and α_5_β_1_ to CTGF. Therefore, one may predict that blocking the β_1_ integrin should have the most potent effect, yet our data showed that its effect was slightly weaker than blocking each individual α integrin. First, the antibodies used do not have the same blocking efficacy. Although these antibodies have been shown to significantly block their respective integrin subunits, they do so with varying efficacy. Second, some integrin receptors, such as α_2_β_1_, have an A-domain in their α subunit that provides the ligand binding site [44,45]. It is for these reasons, that blocking the β_1_ integrin may not have the greatest effect among the antibodies tested in this study. It is interesting to note that there was not a significant difference in the blocking induced by β_1_ versus the individual α integrin subunits, and blocking with a combination of antibodies for two or all three α integrin subunits did not further decrease the adhesion of cells as compared to blocking individual α subunits.

Our results also showed that CTGF is capable of inducing osteoblast adhesion in the presence of two major divalent cations in bone, Mg^++^ and Ca^++^. There are cation binding sites on both α and β subunits of integrin heterodimers and cation binding to these sites can influence integrin-ligand interactions [46]. The synergy between these divalent cations and the relative amounts of each cation are two important factors that have been shown to regulate integrin-ligand binding and subsequent integrin receptor activation [46]. The role of these cations in regulating the adhesive interaction between α_v_β_1_ and CTGF in osteoblasts is of special importance given the dramatic fluctuations in cation concentrations that occur within the bone microenvironment as a result of osteoclast-mediated bone resorption.

Integrins typically bind to their protein ligands in an RGD-dependent fashion which constitutes the major recognition system in cell adhesion [47]. Alternatively, there are an increasing number of interactions between integrins and their ligands that occur in a non-RGD-dependent manner, including the CTGF protein which does not contain an RGD sequence in any of its domains. We screened the fourth domain of the CTGF protein sequence for other known integrin recognition sites: these include the sequence Asp-Gly-Glu-Ala (DGEA) which is present in collagen and is recognized by α_2_β_1_; the sequence Glu-Ile-Leu-Asp-Val (EILDV) which is present in fibronectin and binds to α_4_β_1_; and the sequence Gly-Phe-Hyp-Gly-Glu-Arg (GFOGER) in collagen type I that binds to α_1_β_1_ and α_2_β_1_ integrins [48,49]. Gao and Brigstock identified specific and independent recognition sequences within the fourth domain of CTGF which mediate the adhesion of hepatic stellate and pancreatic stellate cells via the α_v_β_3_ and α_5_β_1_ integrins, respectively, in these two cell types [4,50]. Similar studies are warranted to identify the recognition sequence for osteoblast adhesion to CTGF via the α_v_β_1_ integrin.

Rac is a member of the Rho family of small GTPases. Rho GTPases control signaling pathways that link integrin ligand binding to the assembly and disassembly of actin cytoskeleton [34,50]. Activation of Rac induces the formation of lamellipodia, enhances cell spreading, and regulates formation of focal contacts that grow in size to form focal adhesions as a crucial site for regulation of integrin outside-in signaling pathways [33,34,50,51]. Previous studies have demonstrated that the interaction of integrins with ECM is not sufficient for formation of focal adhesions and downstream signaling activation in absence of functionally active Rho/Rac GTPases [52]. One study on mouse embryonic fibroblasts (MEFs) showed Rac1-null MEFs were defective in lamellipodia formation, cell spreading, cell adhesion to fibronectin, and focal adhesion formation. Rac1 deletion in MEFs caused significant reduction in phosphorylation of signaling molecules including ERK [53]. In MC3T3-E1 cells, a previous study showed that pharmacological inhibition of Rac1 reduced cell adhesion, spreading and proliferation [54]. We showed that osteoblast adhesion to CTGF causes Rac1 activation, an event that is important for actin cytoskeletal reorganization, cell spreading and focal adhesion formation.

Osteoblasts express various integrin receptors that interact with ECM proteins and regulate their growth and differentiation [55–58]. Studies have demonstrated that osteoblast adhesion to matrix proteins, such as type I collagen and fibronectin, through integrin receptors located in focal adhesions is necessary for their differentiation [27,59,60]. The importance of these integrin-ECM interactions has been illustrated in studies demonstrating that disruption of these interactions blocks osteoblast differentiation [61]. Our results showed that osteoblasts cultured on CTGF exhibited enhanced bone nodule formation and matrix mineralization, confirming that this integrin (α_v_β_1_)-ECM (CTGF) interaction stimulates osteoblast growth and differentiation.

In a recent study published by our group [10], we showed that CTGF knockout osteoblasts differentiate similar to wild-type osteoblasts under normal osteogenic conditions. Although it may appear that the results of the previous study contradict the results from our current study, the questions being addressed in these two studies are markedly different. In the Mundy et al. study, differentiation is assessed in primary osteoblasts expressing different levels of CTGF (either normal or absent) when cultured on the same substrate. In the current study, differentiation is being assessed in MC3T3-E1 osteoblasts expressing normal levels of CTGF when cultured on different substrates (immobilized CTGF as compared with positive and negative control substrates). Therefore, the results of the previous study do not preclude or contradict the results of the current investigation.

We did conduct ERK activation experiments at early time points (5 minutes to 2 hours) and found that ERK is rapidly activated upon exposure of cells to a CTGF substrate. However, the primary reason that we assessed activated and total forms of FAK and ERK after 7 days of culture was to determine whether a long-term sustained activation of FAK or ERK could be correlated with the events associated with osteogenic differentiation, since these events occur in days to weeks, not minutes to hours. Our data show that both total and active forms of FAK and ERK are increased. Although the increase in phosphorylated forms of FAK and ERK may be secondary to the long-term increases in the total form of these molecules, this remains an important observation that supports the role of CTGF in eliciting a sustained increase in MAPK (ERK) signaling.

Previous studies on osteoblasts have shown that Runx2 is downstream of the FAK/ERK signaling pathway which is activated by matrix protein-integrin receptor interactions [20,62]. Runx2 induces expression of important bone matrix proteins such as collagen type I, fibronectin, osteopontin, bone sialoprotein and osteocalcin [21–24]. Our data demonstrated that osteoblasts cultured on a CTGF matrix enhances the expression of total and phosphorylated forms of both FAK and ERK, and also increases Runx2 binding to the osteocalcin gene promoter, and the subsequent expression of osteogenic markers regulated by Runx2. These studies provide a mechanistic explanation for the anabolic role of CTGF in bone formation and skeletogenesis that has been alluded to in previous studies [6,7,9,11,12].

## Supporting Information

S1 Fig Flow cytometric analysis of expression of integrin subunits on osteoblasts. Flow cytometry was performed to evaluate the expression of six different integrin subunits (α_v_, α_2_, α_5_, β_1,_ β_3_ or β_5_) on osteoblasts. Cells treated with no antibody, isotype IgG as the primary antibody or secondary antibody only were used as controls.
(TIF)

S2 Fig Cell numbers in osteoblasts cultured on BSA or CTGF coated plates at days 7 and 14. Cell numbers (expressed as relative fluorescent units) were assessed for osteoblasts grown on BSA and CTGF coated plates using a cell proliferation assay at days 7 and 14 of culture. Seeding density at day 0 was identical. N = 6; ns = not significant.
(TIF)

S3 Fig Osteoblast adhesion to CTGF induces short term activation of ERK. Western blot analysis of p-ERK and actin (loading control) from osteoblasts cultured on CTGF for 5 minutes to 2 hours demonstrating maximal activation at 30 minutes post-plating.
(TIF)
