# Supplementary figures and images for: Integrin Mediated Adhesion of Osteoblasts to Connective Tissue Growth Factor (CTGF/CCN2) Induces Cytoskeleton Reorganization and Cell Differentiation
Source: PLoS One. 2015 Feb 25;10(2):e0115325. doi: 10.1371/journal.pone.0115325 (PMC4340870; doi:10.1371/journal.pone.0115325)

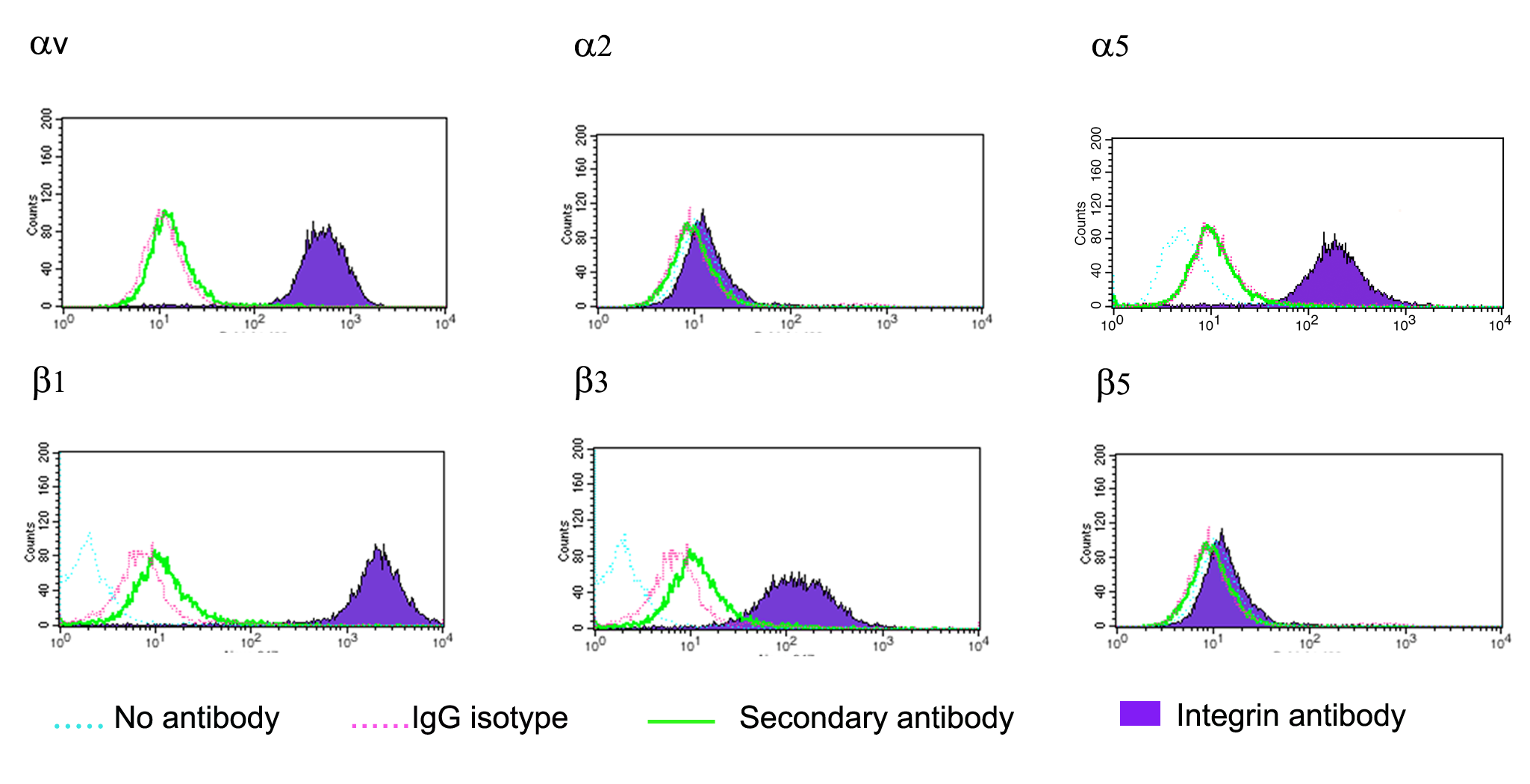

Supplement: S1 Fig — Flow cytometry was performed to evaluate the expression of six different integrin subunits (αv, α2, α5, β1, β3 or β5) on osteoblasts. Cells treated with no antibody, isotype IgG as the primary antibody or secondary antibody only were used as controls. (TIF) [file pone.0115325.s001.tif]

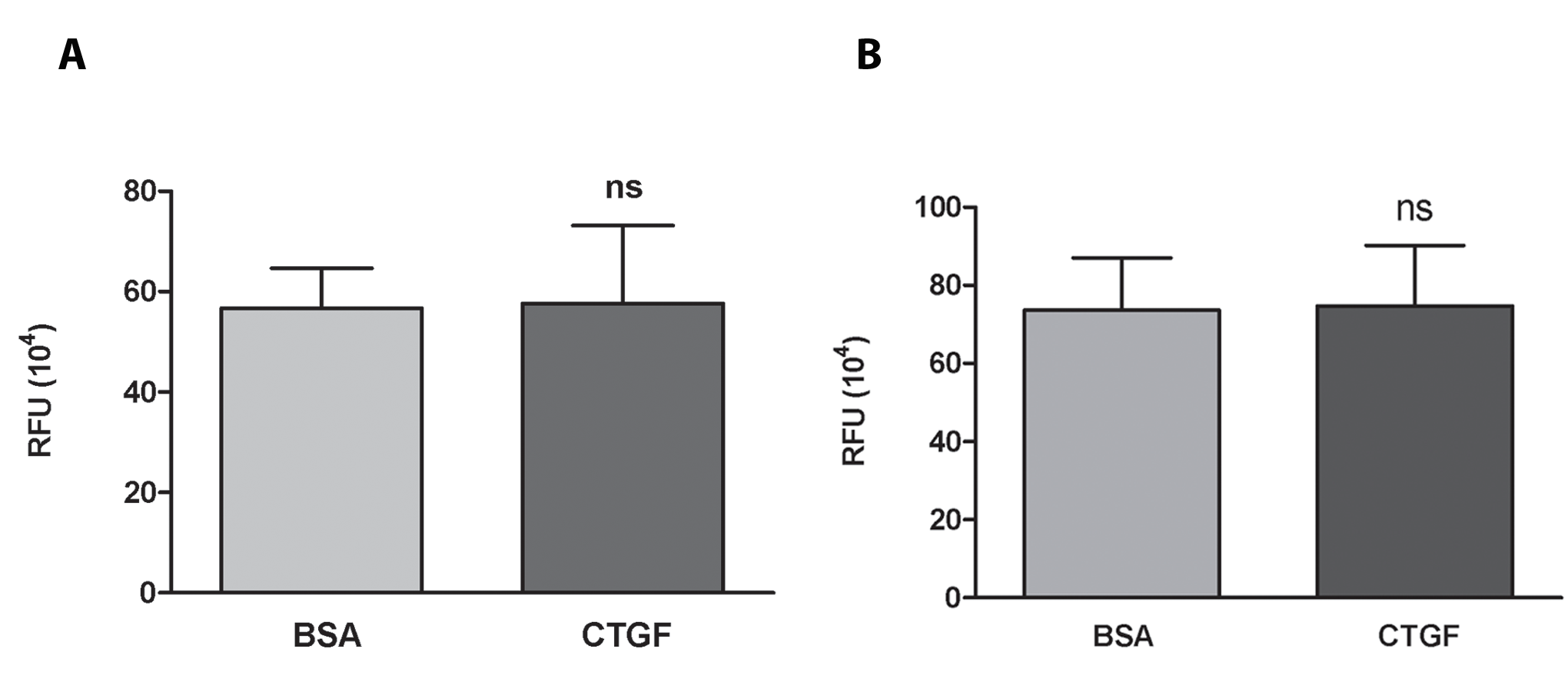

Supplement: S2 Fig — Cell numbers (expressed as relative fluorescent units) were assessed for osteoblasts grown on BSA and CTGF coated plates using a cell proliferation assay at days 7 and 14 of culture. Seeding density at day 0 was identical. N = 6; ns = not significant. (TIF) [file pone.0115325.s002.tif]

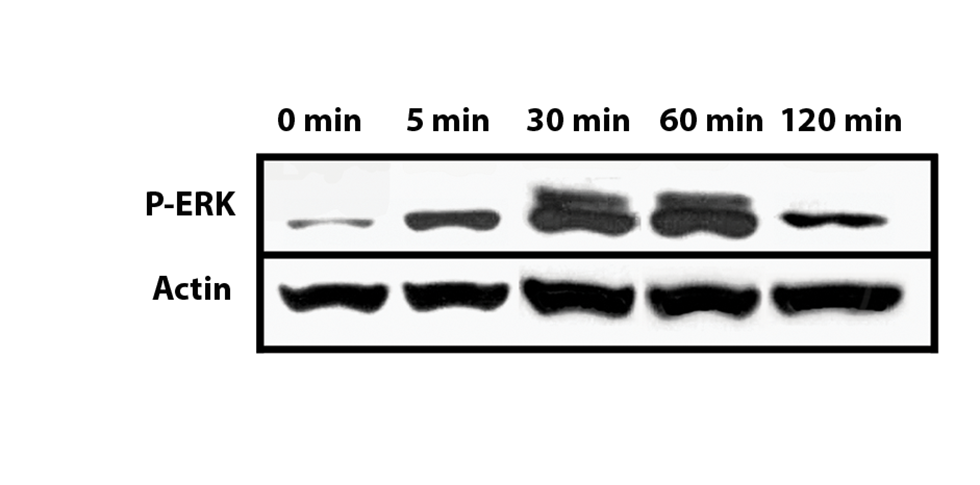

Supplement: S3 Fig — Western blot analysis of p-ERK and actin (loading control) from osteoblasts cultured on CTGF for 5 minutes to 2 hours demonstrating maximal activation at 30 minutes post-plating. (TIF) [file pone.0115325.s003.tif]
